# Early-Age-Related Changes in Proteostasis Augment Immunopathogenesis of Sepsis and Acute Lung Injury

**DOI:** 10.1371/journal.pone.0015480

**Published:** 2010-11-15

**Authors:** Manish Bodas, Taehong Min, Neeraj Vij

**Affiliations:** 1 Department of Pediatric Respiratory Sciences, Johns Hopkins University, Baltimore, Maryland, United States of America; 2 Institute of Clinical and Translational Research, Johns Hopkins University, Baltimore, Maryland, United States of America; University of Alabama-Birmingham, United States of America

## Abstract

**Background:**

The decline of proteasomal activity is known to be associated with the age-related disorders but the early events involved in this process are not apparent. To address this, we investigated the early-age-related (pediatric *vs.* adult) mechanisms that augment immunopathogenesis of sepsis and acute lung injury.

**Methodology/Principal Findings:**

The 3-weeks (pediatric) and 6-months (adult) old C57BL/6 mice were selected as the study groups. Mice were subjected to 1×20 cecal ligation and puncture (CLP) mediated sepsis or intratracheal *Psuedomonas aeruginosa* (*Pa*)-LPS induced acute lung injury (ALI).We observed a significant increase in basal levels of pro-inflammatory cytokine, IL-6 and neutrophil activity marker, myeloperoxidase (MPO) in the adult mice compared to the pediatric indicating the age-related constitutive increase in inflammatory response. Next, we found that age-related decrease in PSMB6 (proteasomal subunit) expression in adult mice results in accumulation of ubiquitinated proteins that triggers the unfolded protein response (UPR). We identified that *Pa*-LPS induced activation of UPR modifier, p97/VCP (valosin-containing protein) in the adult mice lungs correlates with increase in *Pa*-LPS induced NFκB levels. Moreover, we observed a constitutive increase in p-eIF2α indicating a protective ER stress response to accumulation of ubiquitinated-proteins. We used MG-132 treatment of HBE cells as an *in vitro* model to standardize the efficacy of salubrinal (inhibitor of eIF2α de-phosphorylation) in controlling the accumulation of ubiquitinated proteins and the NFκB levels. Finally, we evaluated the therapeutic efficacy of salubrinal to correct proteostasis-imbalance in the adult mice based on its ability to control CLP induced IL-6 secretion or recruitment of pro-inflammatory cells.

**Conclusions/Significance:**

Our data demonstrate the critical role of early-age-related proteostasis-imbalance as a novel mechanism that augments the NFκB mediated inflammation in sepsis and ALI. Moreover, our data suggest the therapeutic efficacy of salubrinal in restraining NFκB mediated inflammation in the adult or older subjects.

## Introduction

Protein homeostasis is regulated by complex cellular processes collectively termed as ‘proteostasis’, which includes protein synthesis, folding, trafficking, disaggregation and degradation [1]. One of the most crucial regulatory components of the proteostasis machinery is the ubiquitin proteasome system (UPS) [2]. The main function of the UPS is to degrade unwanted- or misfolded/damaged- proteins in the cell by protease mediated degradation [3]. Several other functions of this system are now well documented, most importantly, the regulation of important cellular signaling pathways like NFκB activation [4], cell cycle [5] and response to environmental and cellular stress [6], [7]. It also plays a very crucial role in the innate and adaptive immune response, by regulating antigen processing and presentation to the T cells [8]. The importance of UPS in pathogenesis of chronic diseases is evident from its critical role in several debilitating pathological conditions which are the outcome of proteostasis-imbalance [9]. An increase in proteasome activity is associated with sepsis and cancer, where it leads to muscle wasting and inflammation [10], tumor cell survival and metastasis [9]. In contrast, diminished proteasome activity has been related to chronic obstructive pulmonary disease (COPD), cardiac dysfunction, cataract, neurodegenerative and autoimmune diseases and aging [9], [11], [12]. One can easily visualize that most of these pathological conditions are aggravated with advancing age. In fact, COPD, which was thought to be the outcome of long term cigarette smoking, is also described as the disease of accelerated lung aging [11].

One can describe the aging process as a lifelong molecular damage (e.g. accumulation of misfolded proteins) that is aggravated by an age-related decrease in the proteostatic machinery [1], probably at an early phase of aging [13]. The failure of organ or cell maintenance/repair results from the integrated action among genes, environment, and intrinsic defects of the organism that is provoked by constitutive early-age-related changes. Age-related cellular damage often leads to an inflammatory milieu that can exacerbate the existing damage, so that inflammatory and anti-inflammatory factors can play a part in shaping the outcomes of the aging process [14]. The age-related changes result in accumulation of ubiquitinated, oxidized and misfolded proteins [15], [16], [17] that leads to induction of ER stress [18], unfolded protein response (UPR) and inflammation [19], [20]. The accumulation of abnormal or misfolded protein aggregates (aggresomes) is linked to various neurodegenerative disorders that have a higher occurrence with advancing age [21], [22].

The primary mechanism of misfolded protein degradation is the endoplasmic reticulum associated degradation (ERAD) [23], [24]. The UPR modifier, p97/VCP (valosin-containing protein) [25] is known to be associated with key regulatory components of the ERAD pathway [23] that also co-localizes with peri-nuclear protein aggregates, aggresomes [26], [27]. It is involved in both aggresome formation (aggregate formase) as well as clearance of protein aggregates (unfoldase) based on the concentration of aggregation prone-proteins [28]. Since aging is associated with the accumulation of protein aggregates [21], [22], we anticipate that selective inhibition of VCP's aggresome formation function may regress the age-related patho-physiological conditions. Moreover, VCP overexpression is also related to cancer [29], [30], inflammation and NFκB activation [31], [32]. The contribution of VCP to age-related changes in proteostasis and inflammation is although obvious but scarcely investigated. Although decline of proteasomal activity is known to be associated with the age-related disorders but the role of VCP and early events involved in this process are not apparent. To address this, we designed this study to identify the early-age-related (pediatric *vs.* adult) proteostasis mechanisms that augment NFκB mediated inflammation. In addition to focusing on constitutive early-age-related changes, we used both cecal ligation and puncture (CLP) mediated sepsis and *Pseudomonas aeruginosa* (*Pa*)-lipopolysacharride (*Pa*-LPS) induced acute lung injury (ALI) models to verify if these early-age-related changes aggravate the inflammatory pathophysiology.

Sepsis or septic shock is the major risk factor for mortality in the aged population [33]. The mortality rate in the CLP induced septic shock murine model is higher in the older mice [34], owing to a more severe and fatal systemic injury. A recent study by Isaiah R. Turnbull *et al* [35] demonstrates that when injury (CLP) is kept constant, the aged mice develop a more severe inflammatory response that results in higher mortality rates. In contrast, when aged mice receive a less severe injury, they still develop a similar inflammatory response that is comparable to the young mice receiving a more severe injury. These studies suggested that animal's immunopathological response to sepsis determines mortality in aged animals as compared to the young when subjected to similar injury levels but were unable to identify the mechanism for aberrant immune-stress response. Similarly, in *Pa-*LPS induced ALI murine model advancing age is related to an increased pulmonary inflammatory insult [36] and plasma MPO levels. Our study demonstrates the critical role of proteostasis-imbalance as an early age-related change that may be further exacerbated with aging. We found that adult mice are more susceptible to sepsis and ALI as compared to the pediatric group and predict that this may worsen with advancing age resulting in chronic- or fatal- injury.

## Materials and Methods

### Ethics Statement

All animal experiments were carried out in accordance with the Johns Hopkins University (JHU) Animal Care and Use Committee (ACUC) approved protocols.

### Reagents and treatments

The HBE cells were cultured at 37°C with 5% CO_2_ in MEM media supplemented with 10% Fetal Bovine Serum (FBS) and 1% Penicillin, Streptomycin and Amphotericin B (PSA) from Invitrogen. Number of apoptotic cells in longitudinal lung sections of pediatric and adult mice pre- and post- *Pseudomonas aeruginosa* LPS (*Pa*-LPS, Sigma)/CLP treatments were quantified by Dead End™ Fluorogenic TUNEL system (Promega). For *in vitro* experiments, cells were treated with 1 µM MG-132 (Calbiochem) and/or 50 µM Salubrinal (Tocris Bioscience) for indicated time points. Pediatric and adult mice were treated by intratracheal (i.t.) instillation with 1 µg/gm body weight *Pa*-LPS in 100µl total volume of PBS or underwent cecal ligation and puncture (CLP) surgery followed by intra-peritoneal (i.p) treatment with Salubrinal (1 mg/kg body weight). For controls, i.t. mice were compared to 100 µl PBS and CLP group to sham.

### Murine experiments

The weight and sex matched 3 weeks-pediatric and 6 months-adult group (representing young pediatric and 25-year old adult human subjects) of C57BL/6 mice (NCI Animal Production Program) were used to identify early-age- related changes. Mice were housed in controlled environment and pathogen-free conditions. We induced lung injury in these mice by i.t instillation of *Pa*-LPS (1µg/gm bw, in 100 µl PBS) for 24 hours that resulted in approximately 1–1.5 g loss in body weight. The mice were sacrificed after 24 hours of either *Pa*-LPS treatment or CLP (cecal ligation and puncture, described below) surgery and the lungs were harvested in T-PER tissue lysis buffer (Thermo Scientific) supplemented with protease inhibitor cocktail (Sigma) while bronchoalveolar lavage fluid (BALF, isolated using 1 ml PBS) was isolated for IL-6 quantification by standard sandwich ELISA (eBiosciences) following manufacturer's instructions. The serum was also harvested from these mice for quantification of IL-6 (as above) and neutrophil activity using the mouse MPO kit (Hycult Biotechnology) as we previously described [37]. The peritoneal cavity of the mice from CLP group was flushed with 10 ml PBS to isolate peritoneal lavage cells for flow cytometry (described below).

### Cecal ligation and puncture (CLP) surgery

CLP is the most widely used model for experimental polymicrobial sepsis in mice, the procedure was performed as described before [38]. Briefly, mice were anesthetized by 0.1 ml i.p. injection containing ketamine (100 mg/kg) and xylazine (10 mg/kg) in PBS [38]. A midline laparotomy was performed and the cecum was located. The distal 50% of exposed cecum was ligated with 3-0 silk suture and punctured with 1 through and through pass of a 20-gauge needle. The cecum was replaced in the abdomen, and the incision was closed with 3-0 suture. Postoperatively, the animals were resuscitated with 1 ml subcutaneous injection of sterile 0.9% NaCl. After 2 hours of CLP, some groups of mice received a single i.p injection of Salubrinal (1 mg/kg body weight).

### *In vitro* experiments

The HBE cells were treated with MG-132 (1 µM) for 2 hours or overnight. The media was removed and the cells were washed once with sterile PBS. The cells were starved for 30 minutes in methionine**-**cysteine**-**free media (MP Biomedicals) followed by pulse with 250 µCi ^35^S labeled methionine**-**cysteine for indicated time points. The cells were washed in 1× PBS after the indicated time points (pulse) and harvested in M-PER cell lysis buffer (Pierce) supplemented with protease inhibitor cocktail. The total protein lysate was used for ubiquitin immune-precipitation (described below) followed by detection of newly synthesized and metabolically labeled ubiquitin and ubiquitinated proteins by autoradiography [39]. In another experiment, the cells were treated with MG-132 (1 µM) and Salubrinal alone or in combination for 24 hours. Total protein lysates were isolated from cells as described above and changes in levels of ubiquitinated proteins were quantified by immunoblotting.

### Immunoblotting and immunoprecipitation

The mouse lungs from pediatric and adult mice were sonicated (4–5×, 10 sec pulses) in the T-PER tissue lysis buffer or cells were lysed in M-PER, supplemented with protease inhibitor cocktail, centrifuged at 13,000 rpm−4°C for 10 min and the supernatants were collected as total protein lysates. The total protein lysates were immunoblotted for the following antibodies from Santa Cruz Biotechnology (scbt): Ubiquitin (Ub, mouse monoclonal), VCP (rabbit polyclonal), NFκB (rabbit polyclonal), p-eIF2α (rabbit polyclonal), PSMB6 (mouse monoclonal). The anti-β-actin antibody (mouse monoclonal, Sigma) was used as the loading control. The primary antibodies binding was quantified using anti- rabbit or mouse IgG HRP secondary antibodies (Amersham) and Super Signal West Pico Chemiluminescent Substrate kit (Pierce). To evaluate the aggregation of ubiquitinated proteins, we collected the insoluble protein fractions (pellets) by high speed centrifugation (12,000 rpm for 10 mins) of equal amount (500µg) of total protein lysates isolated from pediatric- and adult- murine lungs. These pellets were re-suspended in loading dye containing β-mercaptoethanol (Invitrogen) and run on SDS-PAGE (10%) followed by immunoblotting for Ub and β-actin. For immunoprecipitation experiment, the total protein lysate was used to immunoprecipitate ubiquitin or ubiquitinated proteins using the mouse monoclonal Ub antibody (1 µg, scbt) and protein A/G agarose beads (scbt) as described before [40]. The agarose-ubiquitin conjugate was dissolved in the loading dye containing β-mercaptoethanol and run on SDS-PAGE followed by autoradiography of dried gel (Drygel Sr., Hoefer Scientific Instruments, San Francisco, 80°C for 2 hours) to quantify the changes in metabolically labeled ubiquitinated proteins.

### Immunofluorescence microscopy, TUNEL assay and flow cytometry

The longitudinal tissue sections from murine lungs were immunostained with the primary antibodies (1∶50 to 1∶200 dilution) for Ub (mouse monoclonal, scbt), NFκB (rabbit polyclonal,scbt) and VCP (rabbit polyclonal, scbt) followed by the secondary antibodies (1:200 dilution), using our previously described protocol [37]. The secondary antibodies used were goat anti-rabbit IgG FITC (scbt), donkey anti-mouse Dylight 594 (Jackson ImmunoResearch). Nuclei were detected by Hoechst (Invitrogen) staining while H&E was used to evaluate lung morphology and inflammatory state. The number of apoptotic cells was quantified by Dead End™ Fluorogenic TUNEL system (Promega) using our previously standardized method [41]. Images were captured by Axiovert 200 Carl Zeiss Fluorescence microscope using the Zeiss Axiocam HRC camera and Axiovision software. All fluorescent images were captured at room temperature with air (20× and 40×, fluorescence) as the imaging medium. The magnifications for fluorescence microscope were LD Plan- Achroplan (20×/0.40 Korr Phz) and Neo Fluar (40×/0.6× Phz Korr) with 1.6× optivar. For flow cytometry based analysis, peritoneal lavage cells were subjected to red blood cell lysis using the ACK lysis buffer (Quality Biologicals) followed by 1× PBS wash. The cells were blocked with appropriate sera (goat or donkey) and stained with CD4 (PE-conjugated, rat monoclonal, scbt), Mac-3, (rat monoclonal, Abcam) and NIMP R-14 (rat monoclonal, Abcam) followed by the anti-rat PE labeled secondary antibody. The FIX & PERM®CELL PERMEABILIZATION kit (Invitrogen) was used for intracellular IFNγ-FITC (rat polyclonal, Invitrogen) staining. The stained cells were washed 2-times in FACS buffer and resuspended in 0.1 % paraformaldehyde (USB Corporation). The data was acquired using the BD FACS Caliber instrument and analyzed using the BD Cell Quest software.

### Statistical analysis

Data is represented as the mean ± SEM of at least three experiments, and Student's *t* test and ANOVA were used to determine the statistical significance. The murine microscopy data was analyzed by densitometry (Matlab R2009b, Mathworks Co.) followed spearman's correlation coefficient analysis to calculate the significance among the indicated groups.

## Results

### Early-age-related changes in the proinflammatory response of murine lungs

**Figure 1:**
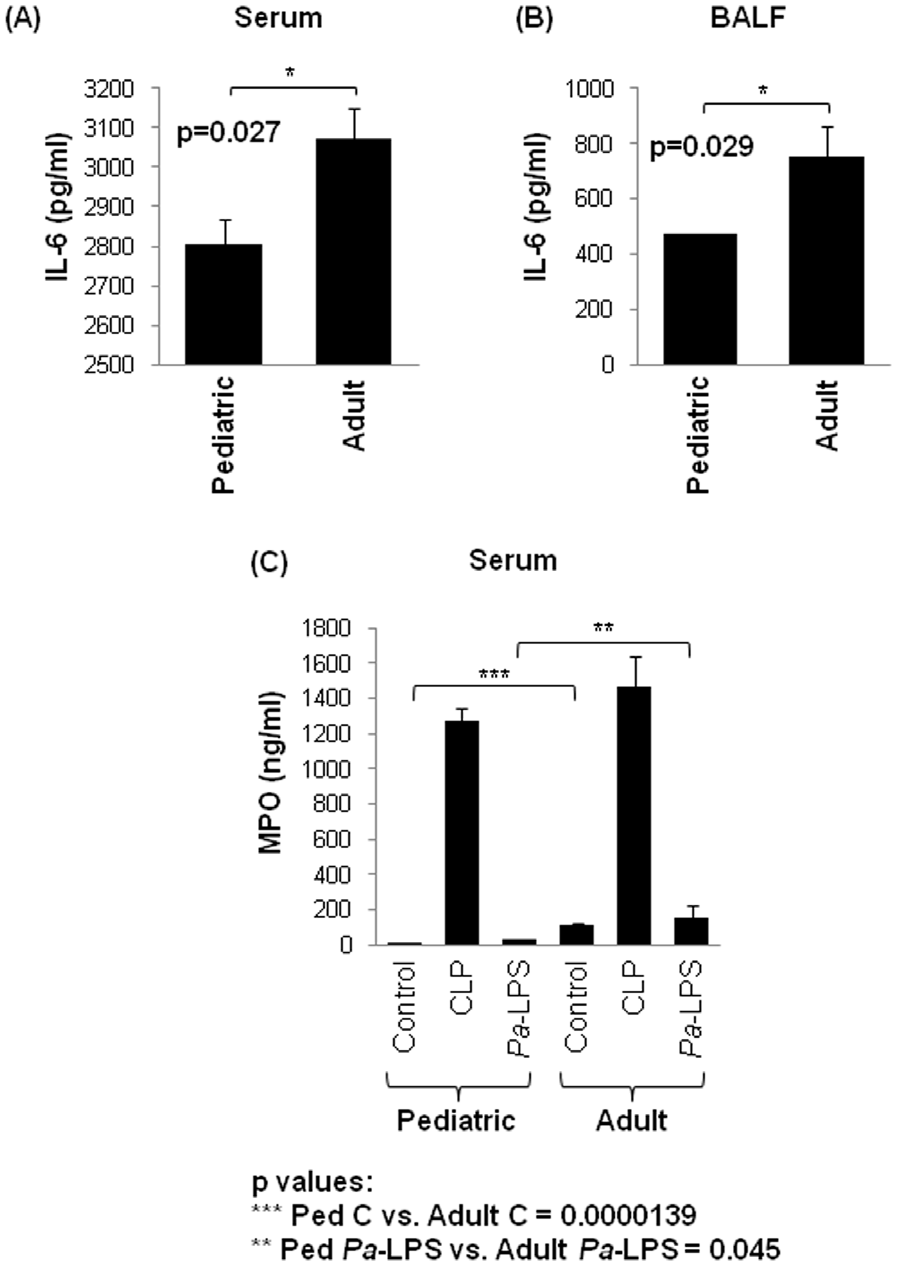
Early-age-related constitutive increase in IL-6 levels and MPO activity. (**A, B**) The constitutive significant (p = 0.027, 0.029) increase in IL-6 levels in the serum and bronchoalveolar lavage fluid (BALF) of the adult (6-month) mice compared to the pediatric (3-weeks) shows an early-age-related change in pro-inflammatory immune response. (**C**) To confirm if intrinsic change in proinflammatory response is an early-age-related event, myeloperoxidase (MPO) activities in serum samples of pediatric- and adult- control (PBS), *Pa*-LPS and CLP, mice were quantified. We found an early-age-related increase in constitutive and *Pa*-LPS induced MPO activity in the adult mice as compared to the pediatric (p values: *** Ped Control vs. Adult Control  =  0.0000139; ** Ped *Pa*-LPS vs. Adult *Pa*-LPS  =  0.045). The data is shown as mean ± SEM of n = 3–4 mice in each group. *The early-age-related changes in the pro-inflammatory response contribute to the immunopathogenesis of sepsis and ALI*.

To evaluate and identify the early-age-related changes that may augment the immunopathogenesis of sepsis or acute lung injury, we compared 3-week (pediatric) to 6-month (adult) old mice group. We observed a significant (p<0.03) constitutive increase in interleukin-6 (IL-6) levels in both serum and bronchoalveolar lavage fluid (BALF) of adult mice as compared to the pediatric (Fig. 1A, B). Next, we verified the early-age-related changes in activity of neutrophil marker, myeloperoxidase (MPO) based on the recent reports demonstrating the higher MPO activity in the serum of elderly humans [42] and rat kidney's [43]. Our data shows a significant increase in constitutive and *Pa*-LPS induced serum-MPO activity (n = 3, Fig. 1C, p<0.05) in the adult mice as compared to the pediatric. We did not observe a similar increase in adult-mice MPO levels after CLP, as compared to the pediatric, although it shows a trend towards an increase. Our data indicate that both IL-6 and MPO activity are constitutively elevated in adult mice as an early-age-related change.

### Early-age-related proteostasis-imbalance augments constitutive NFκB levels

**Figure 2:**
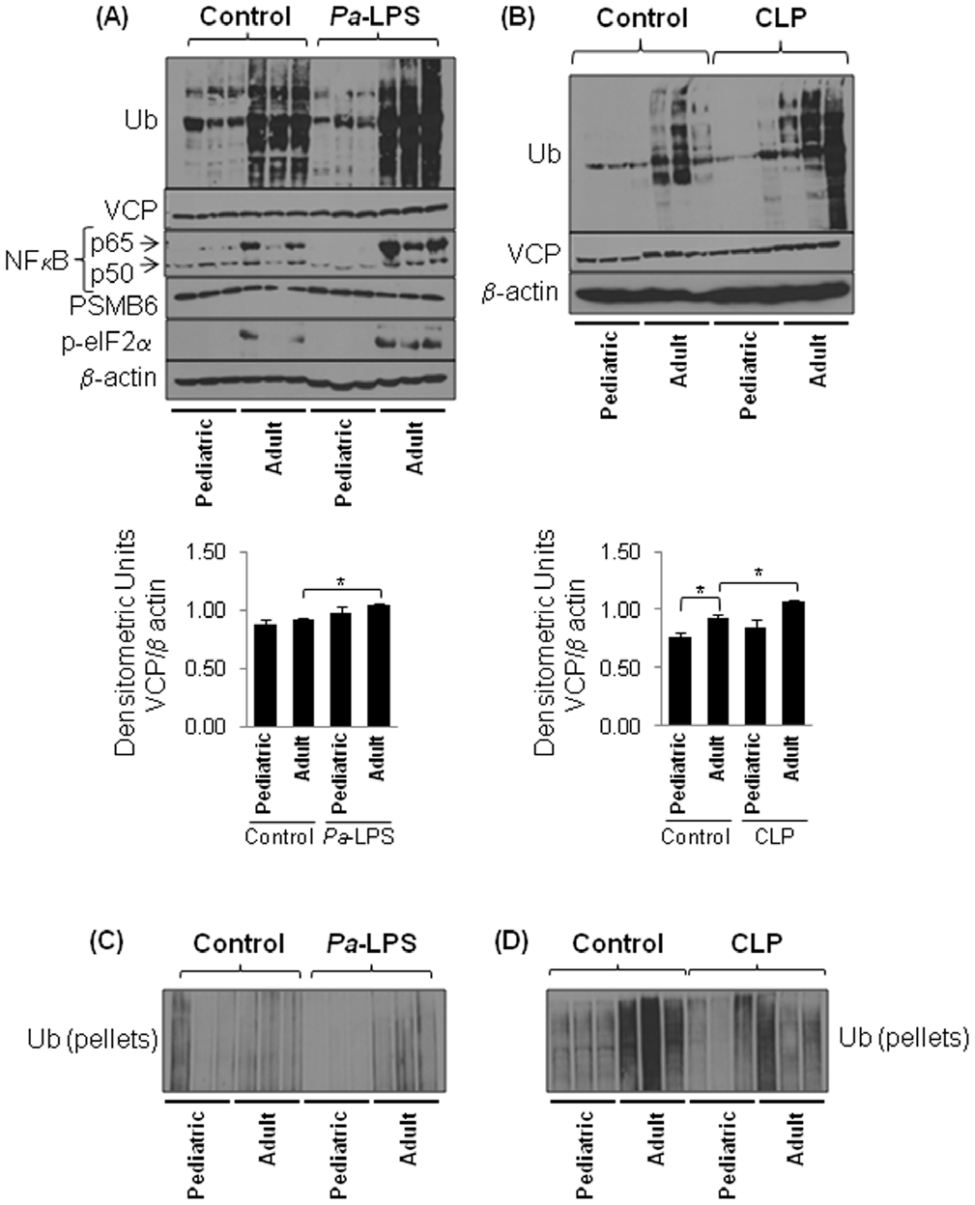
Early-age-related changes in proteostasis induce inflammation. The total protein lung- (A) and liver- (B) lysates from control, CLP or i.t. (1 µg/gm bw) *Pa*-LPS induced murine model (n = 3) were immunoblotted for Ub (ubiquitin), PSMB6 (proteasome-subunit), VCP (UPR), NFκB (Inflammation), p-eIF2α (protective stress response) and β-actin. (**A**) The adult mice lungs show constitutive increase in NFκB and p-eIF2α levels and accumulation of ubiquitinated-proteins while levels of proteasomal subunit, PSMB6 decrease. The *Pa*-LPS further induces the levels of these proteins and VCP in adult mice while PSMB6 is lower indicating that proteostasis-imbalance in adult subjects is a critical early-age-related change that may induce immunopathogenesis of chronic- of fatal- injury in older subjects. (**B**) The liver shows a constitutive increase in VCP expression in the adult mice which correlates with higher accumulation of ubiquitinated proteins. The bottom panel (A & B) shows the densitometric analysis of VCP expression in both lung and liver normalized to β-actin (Mean ± SEM, n = 3, *p<0.05). (**C & D**) Of note here, both lung (C) and liver (D) also show accumulation of ubiquitinated-proteins in the insoluble protein fraction of adult mice as compared to the pediatric (n = 3) but lungs have relatively less accumulation of ubiquitinated proteins in insoluble fraction as compared to the liver. *The early-age-related proteostasis-imbalance induces NFκB mediated inflammatory response*.

To identify the early-age-related changes involved in proteostasis- and inflammatory- imbalance, we used well characterized models of acute lung (intra-tracheal, i.t, *Pa*-LPS instillation) and systemic (cecal ligation and puncture, CLP) [35], [36] inflammation. We found a significant constitutive increase in accumulation of ubiquitinated proteins (Ub) in the adult as compared to the pediatric mice lungs that correlate with higher NFκB and p-eIF2α levels (Fig. 2A, top panel). Moreover, we observed that these changes are amplified in *Pa*-LPS treated adult mice compared to the pediatric. We also observed a correlative significant increase in VCP protein levels in *Pa*-LPS treated adult mice compared to the pediatric (Fig. 2A, top and bottom panels). The induction of VCP expression in chronic inflammatory states like cancer [29], cystic fibrosis [39] and neurodegenerative conditions [44] results in proteostasis-imbalance and pathogenesis of disease. As demonstrated previously for other proteasome subunits [45], we observed a decreased PSMB6 expression in the adult mice lungs compared to the pediatric group (Fig. 2A, top panel) indicative of early-age-related proteostasis-imbalance. We also observed a constitutive change in accumulation of ubiquitinated proteins and VCP expression in the liver of adult mice compared to the pediatric that was further amplified by CLP induced sepsis (Fig. 2B). The aggregation of misfolded- or damaged- proteins is a characteristic of age-related pathogenic conditions [17]. To confirm an early-age-related proteostasis-imbalance we immunoblotted the insoluble protein fractions (pellets) of adult- and pediatric- lung total protein lysates for Ub. Interestingly, the lungs of the older mice have significantly higher levels of ubiquitinated proteins in the soluble fraction (Fig. 2A) as compared to the insoluble (Fig. 2C) indicating accumulation of ubiquitinated proteins in endoplasmic reticulum (ER). In contrast, the liver has most of the ubiquitinated proteins in the insoluble form (aggregates) (Fig. 2D) and not in the soluble fraction (Fig. 2B) suggesting cytosolic aggregation of ubiquitinated proteins (aggresomes). It is possible that liver has higher VCP induction (Fig. 2B, bottom panel) that may lead to aggresome formation, whereas the relatively lower increase in VCP protein levels in lungs may be a response to accumulation of ubiquitinated-proteins in ER as verified by increase in p-eIF2α.

### Age-related proteostasis-imbalance augments inflammation and apoptosis

**Figure 3:**
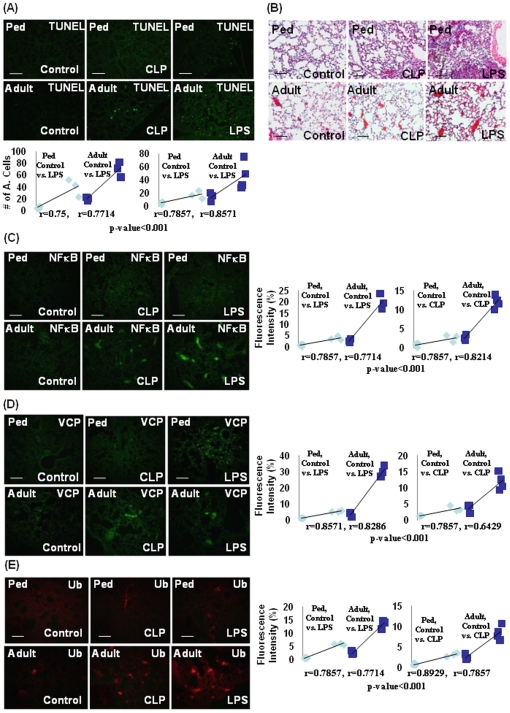
Early-age-related changes in VCP expression correlates with ubiquitin accumulation and hyper-inflammatory response. The longitudinal lungs sections from control, CLP or i.t. *Pa*-LPS (1 µg/gm bw) induced murine model (n = 3–4) were processed for histological evaluation. (**A**) We verified by TUNEL assay that accumulation of damaged- or misfolded- proteins leads to an increase in lung cell apoptosis in the adult mice by quantifying the changes in number of TUNEL positive cells. This is further exacerbated by *Pa*-LPS- or CLP- induced injury, verifying the correlation of ubiquitinated protein accumulation to increased apoptosis. The bottom panel of each staining shows the spearman's correlation coefficient analysis of the number of apoptotic cells in pediatric- and adult- mice (10 uniform representative areas from each mouse). (**B**) The adult mice show a constitutive increase in inflammation as compared to the pediatric group (H&E) that correlates with an increase in NFκB (C) and VCP (D) protein levels. The right panel of each staining shows the spearman's correlation coefficient analysis of fluorescence intensity in pediatric- and adult- mice (10 uniform representative areas from each mouse). (**E**) The *Pa*-LPS and CLP treatments induce the accumulation of ubiquitinated proteins that correlates with further increase in protein levels of NFκB (C) and VCP (D). *Data confirms that early-age-related proteostasis-imbalance augments Pa-LPS or sepsis induced injury*.

The apoptosis of various cell types is known as a critical mediator for pathogenesis of COPD [46], acute lung injury (ALI) [47] and septic shock [48]. Moreover, aging is associated with an augmentation of apoptotic cell death in ALI and COPD [46], [47]. We demonstrate here a significant increase in lung cell apoptosis (TUNEL, Fig. 3A) in the adult mice as compared to the pediatric that was further enhanced by *Pa*-LPS or CLP. The H&E staining of lung sections from control, *Pa*-LPS or CLP mice verify an age-related increase in inflammatory lung phenotype of adult mice as compared to the pediatric (Fig. 3B). Immunofluorescence staining for NFκB and VCP (Fig. 3C & D, left panel) in murine lungs substantiates the immunoblotting data (Fig. 2A). This significant increase (p<0.001) in constitutive as well as *Pa*-LPS or CLP induced NFκB and VCP protein levels (Fig. 3C & D, left panel) in the adult as compared to the pediatric mice also correlates with the ubiquitin accumulation (p<0.001, Fig. 3E, left panel). The statistical analysis of the data, Fig. 3C, D and E is shown in the right panel. Overall, the data verifies that proteostasis-imbalance is an early age-related change that impairs the ability to counteract endotoxin (*Pa*-LPS) or sepsis (CLP) mediated inflammatory insults. We anticipate that this may further exacerbate with aging.

### Salubrinal inhibits accumulation of ubiquitinated proteins and NFκB levels

**Figure 4:**
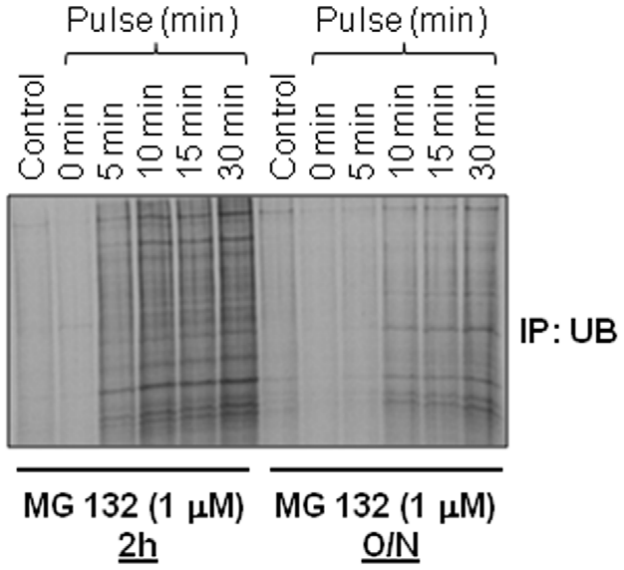
Proteasome inhibition regulates protein turnover rates. The human bronchial epithelial cells (HBE) were treated for 2 hours (2 h, partial) or overnight (O/N, broad) with low dose proteasome inhibitor (MG-132, 1 µM) and accumulation of newly synthesized ubiquitinated protein was monitored over time by metabolic labeling and immunoprecipitation. Lower levels of ubiquitinated proteins in overnight MG132 treated cells during protein synthesis indicate that decreased proteasomal activity inhibits protein synthesis as a feedback inhibition loop. *Lower proteasomal activity inhibits protein turnover rates (proteostasis-imbalance) by inhibiting both protein- synthesis and degradation*.

In order to mimic the aging microenvironment *in vitro*, we treated HBE cells with a low dose of MG-132 (1 µM) for either 2 hours or overnight to read out the effect of lower proteasomal activity on protein turnover rates. We used the metabolic labeling and ubiquitin immunoprecipitation to ‘chase’ the synthesis and accumulation of ubiquitinated proteins. The data shows that overnight treatment with low dose MG-132 diminishes the synthesis of ubiquitinated proteins as compared to the shorter 2 h treatment (Fig. 4). We anticipate that this is a feedback regulatory mechanism to counteract accumulation of ubiquitinated proteins triggered by proteasome inhibition. The data indicate that age-related decrease in proteasomal activity impacts protein turnover rates.

**Figure 5:**
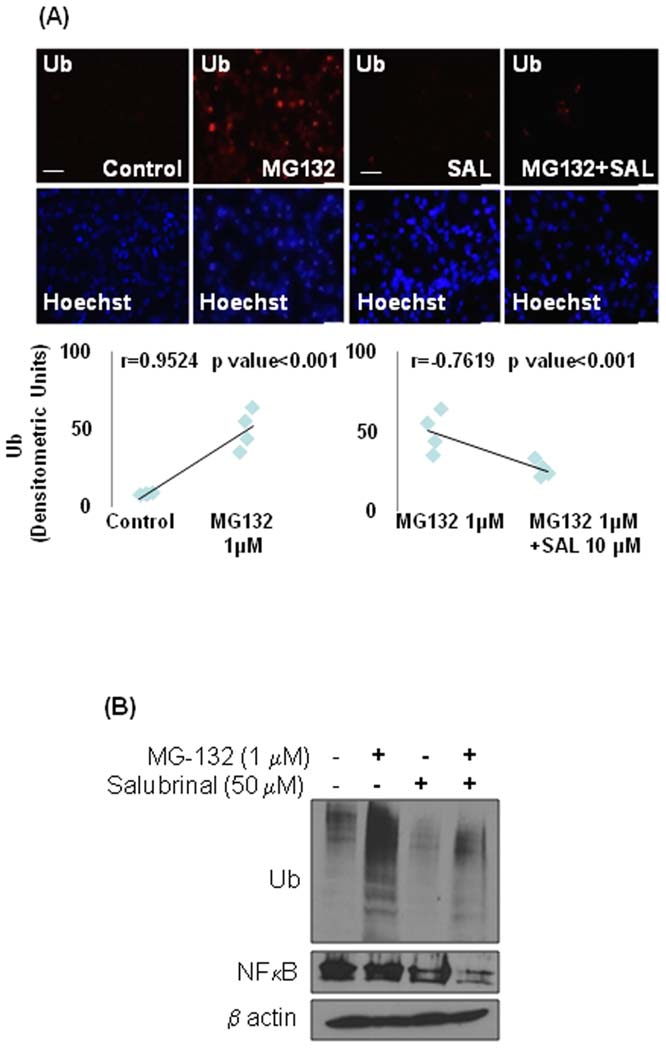
Salubrinal corrects proteostasis-imbalance by inhibiting the accumulation of ubiquitinated proteins. (**A**) The HBE cells were treated with MG-132 (1 µM) for 2 hours followed by treatment with (salubrinal, 50 µM) for another 22 hours and accumulation of ubiquitinated proteins was detected by Ub-immunostaining. The bottom panel shows the spearman's correlation coefficient analysis of fluorescence intensity. Data (n = 4) indicate a significant reduction in MG-132 induced ubiquitinated-protein accumulation by salubrinal, indicating its potential therapeutic use in treating age-related proteostasis-imbalance. (**B**) The HBE cells were treated with MG-132 and/or salubrinal (as in A) and the total protein lysates were immunoblotted for Ub, NFκB and β-actin. Data shows that salubrinal controls ubiquitin accumulation and NFκB induction. *Salubrinal may be effective in controlling age-related proteostasis-imbalance and inflammatory response*.

To standardize the therapeutic strategy for controlling the pathophysiology of age-related proteostasis-imbalance, we induced substantial accumulation of ubiquitinated proteins in HBE cells by treating them with 1 µM MG-132 for 24 hours as detected by immunostaining (Fig. 5A). Since accumulation of ubiquitinated-proteins triggers an ER stress response [18], we controlled the MG-132 induced accumulation of ubiquitinated-proteins by treating these cells with 50 µM salubrinal, a known selective inhibitor of cellular phosphatase complexes that de-phosphorylate eIF2α [49], to clear off protein-aggregation. We verified that salubrinal reduced MG-132 induced ubiquitinated-protein accumulation. Statistical analysis of microscopy data (Fig. 5A, bottom panel) verifies that salubrinal is capable of significantly (p = 0.001) reducing the MG-132 induced ubiquitinated-protein accumulation in addition to protecting cells from ER stress, by interfering with protein folding and/or processing pathways. We confirmed this data by western blotting of total protein lysates from HBE cells treated with the same doses of MG-132 and salubrinal (Fig. 5B). We verified that salubrinal is able to control the NFκB induction (Fig. 5B) as anticipated. Since MG-132 treatment may inhibit the degradation of IκB directly, we did not observe a significant induction of NFκB (Fig. 5B, lane 2) in MG-132 treated cells in spite of ubiquitin accumulation. Our data indicate the therapeutic potential of salubrinal in controlling the proteostasis-imbalance and immunopathogenesis of sepsis and age-related disorders.

### Therapeutic efficacy of proteostasis-regulators in adult murine model of sepsis

**Figure 6:**
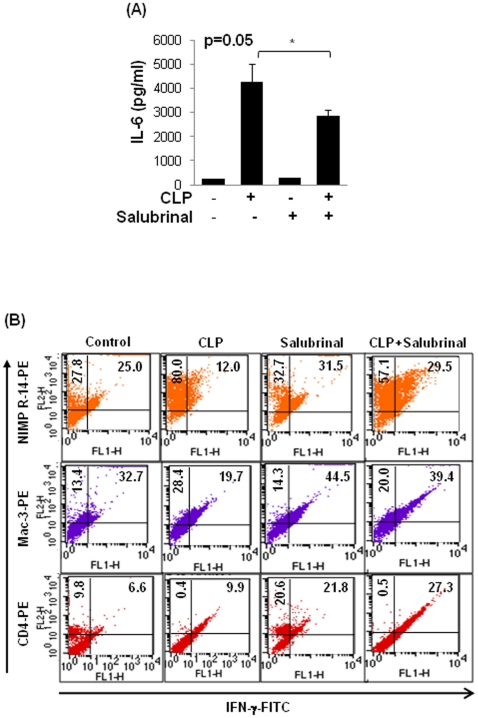
Salubrinal controls CLP induced inflammatory response. The peritoneal lavage from age- and sex- matched control, CLP and/or intraperitoneal (i.p.) salubrinal (1 mg/kg/bw) groups of C57BL6 mice (n = 3–4) were used to quantify changes in IL-6 (A) levels, and number and pro-inflammatory state of inflammatory cells (B). (**A**) Salubrinal is effective in significantly (p = 0.05) controlling the CLP induced pro-inflammatory cytokine, IL-6 levels. (**B**) The flow cytometry analysis shows the number (x-axis) of peritoneal neutrophils (NIMP-R14), macrophages (Mac3) and CD4+ T cells (CD4) isolated from control- and CLP- groups after 24 hrs, with- and without- salubrinal treatment. The pro-inflammatory state of individual cell population was verified by co-staining for intracellular IFNγ (y-axis). Data shows increase in numbers of neutrophils (upper panel) and macrophages (middle panel) with decreasing IFNγ levels after CLP while number of CD4^+^ T cells (lower panel) decrease and have increased IFNγ levels. *Salubrinal is effective in controlling CLP induced inflammatory response*.

The chronic and fatal inflammatory diseases are known to have a more severe outcome with advancing age [11]. An age-dependent increase in ER stress, in wistar rats involves reduced phosphorylation of eIF2α [50]. Hence, we tested the efficacy of salubrinal as an inducer of eIF2α phosphorylation on CLP induced IL-6 secretion and infiltration of pro-inflammatory cells in the adult mice. We found that salubrinal significantly reduces (p = 0.05) CLP induced IL-6 levels in the peritoneal lavage (Fig. 6A) of adult mice. Salubrinal also controls the number of peritoneal pro-inflammatory cells (neutrophils and macrophages) in CLP induced sepsis (C57BL/6 mice, n = 3) (Fig. 6B). A decrease in CLP induced CD4^+^ T cells is previously reported [51], and our data shows that salubrinal is able to restore the decreased number of CD4^+^ T cells. Our *in vivo* (Fig. 6) and *in vitro* (Fig. 5A & B) data suggests that salubrinal can not only reduce ubiquitin-accumulation but may also control the pro-inflammatory response in injury related infection to avoid the immunopathogenesis of chronic or fatal disease in older subjects. Our data warrants further preclinical evaluation of salubrinal in 24-month old mice and Fisher 344 rat aging model (NIA, NIH) based on its ability to control proteostasis-imbalance.

## Discussion

The outcome of any systemic injury is the culmination of genetic, environmental and physiological factors, and all these factors are modulated with increasing age of an organism. We demonstrate that an early-age-related proteostasis-imbalance aggravates the outcome of severe injury which may result in an increased risk of death. A less age difference in the mice for this study was deliberately used to compare and identify the early changes in proteasome mediated protein processing and its outcome on systemic or lung injury with the onset of the aging process. We also demonstrate here the therapeutic potential of salubrinal for treating age-related disorders based on its ability to control MG-132 or CLP induced ubiquitin accumulation, ER stress and NFκB mediated inflammation, the major contributors of age-related pathological conditions.

The age-related decline in proteasome activity is well documented [1], although studies relating its influence on the outcome of severe injury (sepsis/LPS) are scarce [35]. Our data suggest that early-age-related intrinsic proteostasis-imbalance may contribute to the constitutive pro-inflammatory condition [52], Fig. 1A, B)] that can be further exacerbated with infection and/or injury. An early-age-related increase in both constitutive as well as *Pa*-LPS induced neutrophil activity marker, MPO (Fig. 1C) substantiates this conclusion and indicate the critical role of neutrophils in the immunopathogenesis of age-related disorders [11]. The age-related increased in NFκB activity is due to the hyper-phosphorylation of its endogenous inhibitor, IκB, resulting in its retrograde translocation for proteasomal degradation by the valosin-containing protein (VCP) [23]. Our data indicate that VCP activity is higher in the lungs (*Pa*-LPS induced) and liver (constitutive and CLP induced) of adult mice compared to the pediatric as an early-age-related (Fig. 2A, B and Fig. 3D) event that may contribute to the intrinsic as well as *Pa*-LPS/CLP mediated inflammatory signaling, NFκB activity, ER stress and apoptosis. The age-related accumulation of misfolded or damaged proteins in the endoplasmic reticulum (ER) induces apoptosis due to ER stress response [18] while cytosolic aggregation (aggresome) may induce autophagy and apoptosis by ER-independent mechanisms [23]. The ER is a major site of posttranslational protein processing. Equilibrium between the flux of misfolded or damaged proteins entering the ER and the capacity of the organelle to handle its load is maintained by several signaling pathways that together trigger the unfolded protein response (UPR). UPR is initiated in response to an imbalance between protein folding and breakdown capacity or extent of protein overload, a condition leading to ER stress. We demonstrate here that constitutive and *Pa*-LPS induced p-eIF2α levels are up-regulated in the lungs of adult mice (Fig. 2A) as an early marker of proteostasis-imbalance. The increase in p-eIF2α is triggered as a protective stress response to accumulation of ubiquitinated-protein. The p-eIF2α inhibits the overall protein synthesis [53] as a mechanism to combat the accumulation of ubiquitinated-proteins. Similarly, the expression of UPR modifier and proteostasis-regulator, VCP is trigged to control the accumulation of ubiquitinated-proteins. In addition to its UPR modifier function, VCP is a major component of ER-associated protein degradation (ERAD) machinery that is involved in retrograde translocation of misfolded- or damaged- proteins for cytosolic proteasomal degradation [54]. Although VCP levels may be elevated in adults as an protective mechanism to induce the clearance of aggregated proteins [23], [26], it also induces inflammation and apoptosis by mediating the degradation of IκB, an endogenous inhibitor of NFκB. Moreover, age-related changes in VCP levels are known to induce chronic inflammation, pathogenesis of neurodegenerative diseases and cataract [11], [55], [56], [57]. Interestingly, VCP expression is described as a biomarker of Alzheimer's disease [58] which is also an age-related disease [59].

Next, we used proteasome inhibitor- MG-132 treatment of HBE cells as an *in vitro* model to identify that lower proteasomal activity restrain protein turnover rates by inhibiting both protein degradation and synthesis. We identified that overnight incubation of HBE cells with 1 µM MG-132 hampers protein synthesis (Fig. 4) in addition to the accumulation of ubiquitinated proteins (Fig. 5A). This data implies that proteasome inhibition triggers a feedback mechanism by reducing protein synthesis to decrease the load on the proteasome degradation machinery. A similar compensatory mechanism may operate as an early age-related process, where an intrinsic decrease in proteasome activity may diminish protein synthesis, partially restoring the proteostasis imbalance in the organism. Nevertheless, a gradual decrease in the proteasome activity with aging reaches a scenario where this homeostatic mechanism is abrogated leading to various age-related disease conditions. We also used the *in vitro* model (described above) to verify the therapeutic efficacy of salubrinal (a selective inhibitor of eIF2α de-phosphorylation) in controlling protein folding and processing based on its ability to reduce the accumulation of ubiquitinated proteins. The accumulation of ubiquitinated proteins by MG-132 treatment was cleared-off by co-treatment with salubrinal (Fig. 5A, B), which is known to protect cells from UPR by up-regulating and maintaining the levels of p-eIF2α [49]. We anticipate that salubrinal not only inhibits protein synthesis but allows the degradation of aggregated proteins *via* the ubiquitin-proteasome system [60]; a strategy which would be beneficial in the adults and vital for older subjects, where proteasome activity is diminished. Moreover, salubrinal treatment was also able to control the NFκB induction (Fig. 5B). Our data and previous studies [61] suggest that salubrinal controls NFκB mediated inflammatory response by 1) inducing UPR and 2) inhibiting ubiquitin accumulation.

We verify here the therapeutic efficacy of salubrinal to control CLP induced pro-inflammatory cytokine (IL-6) secretion (Fig. 6A). We also demonstrate here that CLP induced infiltration of pro-inflammatory cells in adult murine model of sepsis is controlled by salubrinal (Fig. 6B). Our data suggest that salubrinal may be a useful drug candidate for treating sepsis or ALI in older subjects based on its ability to clear accumulation of ubiquitinated protein and ER stress caused by reduced proteasomal activity. The studies by Isaiah R. Turnbull *et al* indicate that the increased local immune response to sepsis with age is the outcome of a fundamental age-based difference. Our data demonstrates that decrease in proteasomal activity with increased VCP-retrograde translocation activity is an early-age-related change that initiates the accumulation of ubiquitinated proteins inducing NFκB levels, ER stress and apoptosis. Although initial induction of VCP is a cytoprotective response but its prolonged induction in older subjects with decreased proteasomal activity may result in a chronic or fatal injury by chronic NFκB activation and apoptosis.

**Figure 7:**
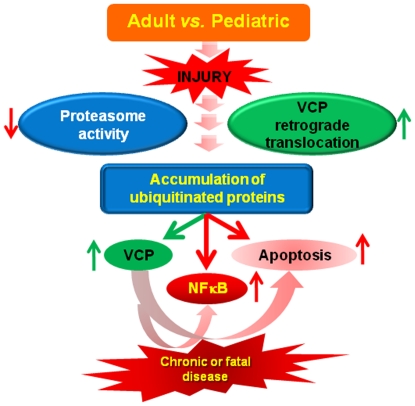
Early-age-related proteostasis-imbalance is critical for immunopathogenesis of chronic or fatal disease. Our data suggest that early-age-dependent decrease in proteasomal activity (PSMB6) results in accumulation of damaged- or misfolded- ubiquitinated proteins leading to an increase in VCP activity as a cytoprotective ER stress response. The sustained increase in VCP expression augments NFκB activation that mediates chronic inflammatory- and apoptotic- signaling. Moreover, accumulation of ubiquitinated proteins has a synergistic effect on these detrimental processes, leading to chronic or potentially fatal injury. Our data suggest the therapeutic potential of salubrinal and selective VCP inhibition in controlling the accumulation of ubiquitinated proteins (proteostasis-imbalance) and NFκB mediated chronic or fatal disease. *We identify here a promising therapeutic strategy to restrain the immunopathogenesis of chronic or fatal injury in older subjects*.

In conclusion, our findings show that an intrinsic deregulation of proteostasis is an early-age-related event that modulates the inflammatory response to *Pa*-LPS/CLP induced injury. We propose salubrinal as a modulator of early-age-related proteostasis-imbalance and its use as a therapeutic strategy to control chronic or fatal injury in the older subjects (Fig. 7). The study warrants further investigation in an appropriate preclinical aging murine- and rat- models as well as young- and old- human subjects.
